# Books and Magazines

**Published:** 1905-11

**Authors:** 


### Books and Magazines.

MANUAL OF OPERATIVE SURGERY. By Jno. Fairbairn Binnie, A. M., C. M. (Aberdeen), Professor of Surgery, Kansas State University, Kansas City, Mo., etc. Second edition revised and enlarged. Philadelphia. P. Blakiston's Son & Co., 1905. Price $3; full morocco, gilt.

The entire first edition of this book was sold out in about six months, necessitating a revision and second edition within a period of nine months. In addition to this record, the book has been adopted as a manual by the United States army and abroad—especially in England—it has been most favorably received.

The present edition has been very carefully revised, pruned in places, enlarged in others, and in every way improved. It contains 567 illustrations, many in colors, and covers 655 twelve-mo. pages. This is an enlargement in both cases. The book is handsomely bound in full flexible morocco, with gilded edges and rounded corners, and sells for $3 net.

TAYLOR ON SEXUAL DISORDERS.—A Practical Treatise on Sexual Disorders in the Male and Female. By Robert W. Taylor, A. M., M. D., Clinical Professor of Genito-Urinary and Venereal Diseases in the College of Physicians and Surgeons (Columbia University), New York. New (third) edition, enlarged and thoroughly revised. In one octavo volume of 575 pages, with 130 engravings and 16 colored plates. Cloth, $3.00 net. Lea Brothers & Co., Philadelphia and New York, 1905.

The demand for three large editions of Dr. Taylor's excellent work in less than five years indicates the position of authority in its field which it has won. The volume covers the sexual disorders of both sexes fully in all their bearings. In this edition the chapter on the Anatomy and Physiology of the sexual apparatus has been very much amplified and many new illustrations have been added. Throughout the work diseases of this nature have been treated in careful detail, many typical cases being given, thus facilitating diagnosis and rendering most practical aid in questions of treatment both medical and surgical. The text has been thoroughly revised to date, and the sections which treat of the sexual disorders in women have been greatly enlarged. Four completely new chapters have been added and a number of new illustrations, mostly original, will, it is believed, render this valuable work still more useful to the specialist and general practitioner. Dr. Taylor's work is practically the only one in English in its exact field, and its great practical worth is clearly reflected in the demand which affords such frequent opportunities for revision.

---

A TEXT-BOOK OF CHEMISTRY.—For the use of Students and Practitioners of Medicine, Dentistry and Pharmacy. By William Russell Jones, M. D., Ph. D., Professor of Medical Chemistry and Toxicology and Lecturer on Medical Diagnosis in the University College of Medicine, etc., Richmond, Va. Illustrated. Philadelphia. P. Blakiston's Son & Co., 1905. Price $2.50 net, 460 pages, cloth.

This is an excellent work for students. Technicalities are avoided as far as possible, and the language used is of easy comprehension by the beginner. It is a valuable text-book. The introduction deals with the Properties of Matter. Part I. deals with Physics, considering heat, light, electrical energy, etc. Part II. takes up Chemical Philosophy, dealing with the atomic theory, elements and compounds, atomic and molecular weights, the elements, etc. Part III. considers Inorganic Chemistry. Part IV. Organic Chemistry. Part V. Quantitative Analysis, and Part VI. Physiological Chemistry.

---

OPHTHALMIC NEURO-MYOLOGY.—A Study of the Normal and Abnormal Actions of the Ocular Muscles from the Brain Side of the Question. By G. C. Savage, M. D., Professor of Ophthalmology in the Medical Department of Vanderbilt University. Author of "New Truths in Ophthalmology" (1903) and "Ophthalmic Myology," 1902. Ex-President Nashville Academy of Medicine; Ex-President Tennessee State Medical Association. Thirty-nine full page plates and twelve illustrative figures. Published by the author. Cloth bound, 220 pages. Price not stated.

The character of the work is fully indicated in the title. Dr. Savage is an authority of recognized ability.

---

GRAY'S ANATOMY.—Messrs. Lea Brothers & Co. have pleasure in announcing a new edition of Gray's Anatomy, to be published about midsummer, and embodying nearly two years of labor on the part of the editor, J. Chalmers DaCosta, M. D., of Philadelphia, and a corps of special assistants.

Commensurately with the importance of the largest selling medical work ever published, this new edition will present a revision so thorough and searching that the entire book has been reset in new type. In addition to the changes necessary to bring it abreast of the modern knowledge of its subject, several important alterations have been made with the view of adapting it still more closely to present-day teaching methods, and in fact to anticipate the trend of anatomical work and study.

Thus, while the older nomenclature is used, the new names (B. N. A.) follow in brackets; the section on Embryology and histology at the back of the present "Gray" has been distributed throughout the new edition in the shape of embryological, histological and biological references and paragraphs bearing directly on the part under consideration, thus contributing to a better and easier understanding.

The illustrations have come in for their full share of the general revision, so that at this writing more than 400 new and elaborate engravings in black and colors have been prepared. "Gray" has always been noted for its richness of illustration, but the new edition far exceeds anything that has hitherto been attempted.

No medical text-book has ever approached "Gray" in sturdy longevity and accumulating strength. Notwithstanding the many would-be competitors which during nearly fifty years have periodically appeared and endeavored to share its ever-increasing popularity, this wonderful creation of a genius who lived barely long enough to realize that his work was done—how well he never knew—goes on and on, each succeeding year bringing new friends and strengthening the fealty of the old.

The editor and publishers have spared neither labor nor expense to keep "Gray" at the forefront of anatomical knowledge, and there seems to be no reason to doubt that its next fifty years will pass as smoothly and as successfully as have those past.

---

Surgery, Gynecology and Obstetrics. We have received Vol. 1, No. 1 of a new monthly by the above title. It is published at Chicago, and the managing editor is Dr. Franklin H. Martin. Among the contributors to No. 1 are Senn, Montgomery, Van Hook, Webster, Coe and Dudley. One hundred and twelve double column pages of reading matter by well known and high authorities. Subscription price $5. This publication fulfills the mission that the Octopus ought to fill, but doesn't. I hope it will succeed.

---

Green's Pathology.—Tenth edition. A Text-book of Pathology and Pathological Anatomy. By T. Henry Green, M. D., F. A. C. P., Consulting Physician to Charing Cross Hospital, London. New (tenth) edition. Thoroughly revised by W. Cecil Bosanquet, A. M., M. D., F. R. C. P., Assistant Physician to Charing Cross Hospital. Octavo, 606 pages, 348 engravings and a colored plate. Cloth, $2.75, net. Lea Brothers & Co., Publishers, Philadelphia and New York, 1905.

The vigor and vitality of Green's Pathology are excellent evidences of qualities which have won the favor of students, professors and practitioners alike. A text-book which has reached its tenth edition stands in no need of an introduction. This work has been from its first edition a simple, clear and adequate presentation of pathology and pathological anatomy—the foundation of all medicine. The subject of the work has undergone many changes since Green was first issued. Pathology is by no means stagnant. In fact, it is doubtful whether any branch of medicine has undergone so rapid and so transforming a growth during recent years. The demand for Green has made necessary very frequent editions. Each edition represents a thorough revision, but none, perhaps, so thoroughgoing as the present. The pages of this work have, therefore, always been consulted for the recent advances and condition of its science, and the diction of the work is so clear, so directly to the point, so easy of understanding, that the popularity of Green throughout the student world, and equally as a quick reference for the busy practitioner, is not to be wondered at.

---

A Text-Book of Clinical Diagnosis.—By Laboratory Methods. For the use of Students, Practitioners, and Laboratory Workers. By L. Napoleon Boston, A. M., M. D., Associate in Medicine and Director of the Clinical Laboratories at the Medico-Chirurgical College, Philadelphia. Second edition, revised and enlarged. Octavo of 563 pages, with 330 illustrations, including 34 plates, many in colors. Philadelphia and London. W. B. Saunders & Company, 1905. Cloth, $4.00 net; Sheep or Half Morocco, $5.00 net.

It must, indeed, be a great gratification to an author when two editions of his work are required in one year. From such a reception it is evident that Dr. Boston's Clinical Diagnosis fills a demand. In this new second edition many new subjects have been added, including Biff's New Hemogelometer, Ficker's Reaction, an illustrated description of the Leishman-Donovan Bodies, Ravold's Test for Albumin, Cammidge's Test for Glycerin, and Cipollino's Test. The subjects of cystodiagnosis and inoscopy are given more extended consideration, the practical usefulness of these methods have been clearly demonstrated. Throughout the text it has evidently been Dr. Boston's aim to emphasize in progressive steps the various procedures of clinical technic, illustrating such steps whenever possible. An unusual amount of space is given to the consideration of animal parasites, malarial and other blood parasites, skin diseases, transudates and exudates, and the secretions of the eyes and of the ears.

---

Modern Clinical Medicine. This work is translated from the German, and will consist of three large volumes. The first of the series is received. It is entitled "Infectious Diseases," edited by J. C. Wilson, M. D., Professor of Medicine at the Jefferson Medical College of Philadelphia. Price, cloth, $6.00. New York: D. Appleton & Co. 1905.

The translation is made under the editorial supervision of Dr. Julius L. Salinger, of Philadelphia, from "Die Deutsche Klinik." The articles in "Die Deutsche Klinik" are written by the foremost clinicians in Germany, men who are well known authorities on the subjects of which they write. Professors Leyden and Klemperer are the German editors. The contributors are such well-known specialists as Leube, Ewald, Boas, Baginsky, Nothnagel, Liebermester, Eichhorst, Strumpell, Curschmann, Jurgensen, Ehrlich, Grawitz, Binz, Gerhardt, Hoffa, Loeffler, Senator, Krafft-Ebing, Ortner and Kaposi.

The American editors have made many annotations, and at times written entire chapters upon diseases which were not included in the German.

"Infectious Diseases" is a volume of 925 pages, including the index, which is unusually complete. The work contains many illustrations in black and white and two colored plates. The entire field of infectious diseases is covered fully and practically. There is nothing in this work that is unnecessary, but everything which is essential. There are chapters on typhoid and typhus fever, diphtheria, measles, scarlet fever, smallpox, cholera, erysipelas, etc.

The volumes in "Modern Clinical Medicine" will be sold separately.

The second volume in this series, which is now in preparation, is

"Constitutional Diseases and Diseases of the Blood." The third in the series will be "Diseases of the Digestive Tract."

